# Nutraceuticals and mitochondrial oxidative stress: bridging the gap in the management of bronchial asthma

**DOI:** 10.1007/s11356-022-21454-w

**Published:** 2022-07-07

**Authors:** Venkata Sita Rama Raju Allam, Keshav Raj Paudel, Gaurav Gupta, Sachin Kumar Singh, Sukriti Vishwas, Monica Gulati, Saurabh Gupta, M. V. N. L. Chaitanya, Niraj Kumar Jha, Piyush Kumar Gupta, Vyoma K. Patel, Gang Liu, Mohammad Amjad Kamal, Philip M. Hansbro, Brian Gregory George Oliver, Dinesh Kumar Chellappan, Kamal Dua

**Affiliations:** 1 Department of Medical Biochemistry and Microbiology, Uppsala University, Uppsala, Sweden; 2 Centre for Inflammation, Centenary Institute and University of Technology Sydney, School of Life Sciences, Faculty of Science, Sydney, NSW 2007 Australia; 3 School of Pharmacy, Suresh Gyan Vihar University, Jagatpura, Jaipur, India; 4 Department of Pharmacology, Saveetha Dental College, Saveetha Institute of Medical and Technical Sciences, Saveetha University, Chennai, India; 5 Uttaranchal Institute of Pharmaceutical Sciences, Uttaranchal University, Dehradun, India; 6 School of Pharmaceutical Sciences, Lovely Professional University, Phagwara, Punjab India; 7 Chitkara College of Pharmacy, Chitkara University, Punjab, India; 8 Department of Biotechnology, School of Engineering & Technology (SET), Sharda University, Greater Noida, Uttar Pradesh India; 9 Department of Biotechnology, School of Applied & Life Sciences (SALS), Uttaranchal University, Dehradun, 248007 India; 10 Department of Life Sciences, School of Basic Sciences and Research (SBSR), Sharda University, Greater Noida, Uttar Pradesh Australia; 11 School of Clinical Medicine, Faculty of Medicine and Health, University of New South Wales, Sydney, NSW 2052 Australia; 12 King Fahd Medical Research Center, King Abdulaziz University, P. O. Box 80216, Jeddah, 21589 Saudi Arabia; 13 Institutes for Systems Genetics, Frontiers Science Center for Disease related Molecular Network, West China Hospital, Sichuan University, Chengdu, China; 14 Enzymoics, Novel Global Community Educational Foundation, 7 Peterlee Place, Hebersham, NSW 2770 Australia; 15 Department of Pharmacy, Faculty of Allied Health Sciences, Daffodil International University, Dhaka, 1207 Bangladesh; 16 School of Life Sciences, Faculty of Science, University of Technology Sydney, Ultimo, NSW Australia; 17 Woolcock Institute of Medical Research, University of Sydney, Sydney, NSW Australia; 18 Department of Life Sciences, School of Pharmacy, International Medical University, Bukit Jalil, Kuala Lumpur, 57000 Malaysia; 19 Discipline of Pharmacy, Graduate School of Health, University of Technology Sydney, P.O. Box: 123 Broadway, Ultimo, NSW 2007 Australia; 20 Faculty of Health, Australian Research Centre in Complementary and Integrative Medicine, University of Technology Sydney, Ultimo, NSW 2007 Australia

**Keywords:** Nutraceuticals, Mitochondria, Oxidative stress, Asthma, Inflammation, Therapeutics

## Abstract

Asthma is a chronic inflammatory disease primarily characterized by inflammation and reversible bronchoconstriction. It is currently one of the leading causes of morbidity and mortality in the world. Oxidative stress further complicates the pathology of the disease. The current treatment strategies for asthma mainly involve the use of anti-inflammatory agents and bronchodilators. However, long-term usage of such medications is associated with severe adverse effects and complications. Hence, there is an urgent need to develop newer, novel, and safe treatment modalities for the management of asthma. This has therefore prompted further investigations and detailed research to identify and develop novel therapeutic interventions from potent untapped resources. This review focuses on the significance of oxidative stressors that are primarily derived from both mitochondrial and non-mitochondrial sources in initiating the clinical features of asthma. The review also discusses the biological scavenging system of the body and factors that may lead to its malfunction which could result in altered states. Furthermore, the review provides a detailed insight into the therapeutic role of nutraceuticals as an effective strategy to attenuate the deleterious effects of oxidative stress and may be used in the mitigation of the cardinal features of bronchial asthma.

## Introduction

Asthma is a multifaceted heterogenous chronic respiratory disease associated with various phenotypes and endotypes, sharing common characteristic features like inflammation and reversible airway obstruction (Mehta et al. 2021d; Ray et al. 2016). Asthma is a non-communicable chronic inflammatory airway disease and is one of the leading causes of morbidity and mortality affecting 300–400 million individuals worldwide. The disease is responsible for around 461,000 deaths each year (Anonymous 2020). Asthma is mainly driven by the attribution of both environmental allergens (including bushfires) and epigenetic changes which result in episodic or persistent respiratory symptoms such as cough, shortness of breath, wheezing, tightness in chest, and variable degrees of airflow limitation due to bronchoconstriction, airway inflammation, and increased mucus secretion (Dharwal et al. 2020; Hinge et al. 2020; Papi et al. 2020).

Asthma is classified into various phenotypes and endotypes depending on various factors including inflammatory status, onset and severity of the disease, and molecular mechanisms involved in the disease pathogenesis. Based on the inflammation, asthma is classified into type 2 asthma and non-type 2 asthma endotypes (Woodruff et al. 2009) and eosinophilic, neutrophilic, mixed granulocytic, and paucigranulocytic phenotypes. Similarly, based on the molecular mechanism of the disease, inflammatory patterns, onset and severity of the disease, Kaur and Chupp (2019), Kuruvilla et al. (2019), and Pembrey et al. (2018), have identified various phenotypes including early-onset allergic asthma, early-onset allergic moderate-to-severe remodelled asthma, late-onset nonallergic eosinophilic asthma, and late-onset nonallergic non-eosinophilic asthma further helps in improving the clinician approach for a better characterization and treatment.

Although genetic variations is considered one of the main factors involved in the asthma progression that can be studied using various methods like candidate gene approach, genome-wide association studies, and gene interaction studies, other non-genetical factors like environmental triggers are also involved in disease pathogenesis. The main trigger for the asthma development is exposure to various allergens; however, other environmental irritants including exposure ambient traffic pollution, polycyclic aromatic hydrocarbons, exercise, diet, and industrial and occupational exposures are also involved in the disease progression (Blumenthal 2012). Many studies have depicted the impact on the exposure of both the outdoor and indoor environmental irritants in exacerbating and triggering asthma during the early life mainly through reprogramming the lung architecture which leads to the generation of immature lung that is more susceptible to the asthma risk in the late life (Ho 2010).

The initiation and progression of the asthma disease is mainly involved with activation of various inflammatory pathways combining with altered airway homeostasis of the tissues and cells involved in the lung physiology. The progression of the asthma is mainly characterized with two phases: an early phase and a late phase. The early phase in the presence of environmental allergens is initiated by the IgE immunoglobulins that are released by the sensitized plasma cells which then bind to various immune resident cells including mast cells and basophils (Picado 1992). The mast cells in the presence of the immunoglobulins further get activated and degranulated to release various inflammatory mediators, proteases, and bronchoconstrictor agents including IL-6, IL-33, chymase, tryptase, carboxypeptidase 3, and histamine to initiate the late phase of the disease (Allam et al. 2021; Pejler 2019). The initiation of late phase further promotes the T-helper cell differentiation and proliferation into Th1, Th2, and Th17 cells (Pradalier 1993). These polarized Th cells release various chemotactic factors like IL-4, IL-5, IL-13, IL-17, and growth factors to promote eosinophil and neutrophil chemotaxis to the lung site and attributes various disease-associated factors including smooth muscle thickening, airway remodelling, airway constriction, fibrosis, and airway hyperresponsiveness (Davoine and Lacy 2014; Shastri et al. 2021; Zhu et al. 2020).

Current therapy for asthma treatment mainly involved in the usage of bronchodilators and both beta 2 (β_2_) adrenergic agonists and anticholinergic agents that relax the airway smooth muscle to attenuate the bronchoconstriction, and anti-inflammatory drugs including corticosteroids and methylxanthines to suppress the airway-associated inflammation (Barnes 2011; Barnes 2016; Gross and Barnes 2017; Papi et al. 2020). However, the usage of the steroids is limited especially in treating severe asthma and neutrophilic and paucigranulocytic asthmatics who responds poorly to the conventional steroid therapy and also leads to corticosteroid resistance (Barnes 2017; Paudel et al. 2020a). In addition, long-term use of steroids and bronchodilators is also associated with several side effects including weight gain, hyperglycemia, cataract formation, glaucoma, increased body weight, and gastrointestinal bleeding, osteoporosis, tachycardia, and tremors (Allam et al. 2021). Also, long-term usage of bronchodilators leads to various adverse effects including dry mouth, pupillary dilation, blurred vision, acute glaucoma, and cognitive dysfunction (Gupta and O’Mahony 2008). Various novel anti-inflammatory therapies including methylxanthines, biologics, and kinase inhibitors have also been investigated in the treatment of asthma. However, factors including narrow therapeutic index of the drugs, unwanted adverse effects associated with multiple kinase inhibition, and high cost of the biologics have largely restricted their use clinically. Another issue of using the modern anti-asthmatic drugs is limited to their efficacy as they are effective only if administered particularly at the time when maintaining the chronotherapy, as the onset of symptoms and exacerbation varies between the patients (Paudel et al. 2021). Therefore, a better understanding of the mechanistic pathways (Mehta et al. 2020a, b) and investigating alternate novel mechanisms involved in the pathogenesis of asthma are highly needed to develop alternate therapies and new methods of advance drug delivery (example: liquid crystalline nanoparticles, decoy oligonucleotide, extracellular vesicles, and polysaccharides) with minimal side effects and enhanced efficacy (Chan et al. 2021a; Manandhar et al. 2022; Mehta et al. 2021b; Prasher et al. 2021).

Oxidative stress is a widely known scientific term and it is relevant to almost all human diseases as generation of reactive oxygen species (ROS) affects every organ such as lungs and cardiovascular system (Mehta et al. 2021c; Nucera et al. 2022; Panth et al. 2016b). The ROS generated during oxidative stress is a potent trigger of cellular senescence whereby it irreversibly limits the cell proliferation and these senescent cells secretes various senescence-associated secretory phenotype that further induced the senescence of adjacent cells. As the trigger of asthma such as allergen and environmental pollutants is already established as a stimulant of oxidative stress, the inhalation of these noxious agent is closely associated with senescence of airway cells (Wang et al. 2020b). Apart from senescence, increased ROS production from immune cells such as neutrophil and macrophages in asthma patients is also associated with an increase in inflammasome activation (example NLRP3) that further exacerbates airway inflammation (Simpson et al. 2014). Studies have shown that inflammasome-mediated IL-1β responses may play a role in the pathogenesis of neutrophilic asthma (Kim et al. 2015). Research also suggests that the toll-like receptor (TLR)-4-associated p38 mitogen-activated protein kinase (MAPK) signaling pathway is involved in autophagy and oxidative stress (Wang et al. 2020a). The vital trilateral linkage between TLR, innate immunity, and lung disease is helpful to further understand the asthma pathophysiology (Patel et al. 2022). As such, targeting senescence and inflammasomes in asthmatic (clinical study) or pre-clinical animal model (mice, rats) by advance drug delivery system such as nanotherapeutics could be a promising approach for the management of asthma (Devkota et al. 2021b; Khursheed et al. 2022; Paudel et al. 2022a; Tan et al. 2022). To study the pathophysiology of asthma and test various drugs in pre-clinical setting, experimental animal models of asthma are very useful. This model develops characteristic feature of human asthma by exposing animals such as rats and mice with allergen such as ovalbumin (OVA), house dust mite (HDM), cockroach allergen, air pollution, and biomass smoke (Gold et al. 2016; Hirota et al. 2015; Liu et al. 2017).

As discussed earlier, asthma is a multifaceted disease associated with the involvement and activation of various biological and molecular pathways in the disease progression. Despite the activation of the inflammatory pathways, asthma pathogenesis is also progressed by the altered homeostasis of the various cellular mechanisms which includes autophagy dysregulation (Theofani and Xanthou 2021), increased endoplasmic stress (Miao et al. 2020), and mitochondrial stress (Sachdeva et al. 2019) further causes cellular dysfunction and cell death. The altered homeostasis processes also creates an imbalance between oxidant and antioxidant system in the lungs and results in the abnormal rise of the oxidative radicals in the lungs and increased oxidative stress (Erzurum 2016). Increased oxidative stress in the asthma pathogenesis is mainly associated with the response of both the exposure of external stimulants including the environmental pollutants, allergens, and generation of endogenous oxidative radicals due to imbalance in the oxidant-antioxidant system (Sahiner et al. 2018). The increased oxidative stress along with the response to the environmental irritants exposure further augments various disease characteristics of asthma including initiation and progression of inflammation, airway hyperresponsiveness, and airway obstruction (Kirkham and Rahman 2006). The oxidative stress allied with the increased endogenous ROS like superoxide anion (O_2_^−)^, hydrogen peroxide (H_2_O_2_), and hydroxyl radicals (OH^−^) are mainly generated either due to mitochondria dysfunction or by the activation of the various enzyme oxidases including NADPH oxidase, P450 mono-oxygenase, cyclooxygenase, indolediamine dioxygenase, xanthine oxidase, and the Rho kinases (Kirkham and Rahman 2006). A lot of studies have examined and reported previously regarding the involvement of the oxidative stress in the initiation and progression of asthma (de Groot et al. 2019; Jesenak et al. 2017; Mishra et al. 2018; Sahiner et al. 2018).

Nutraceuticals are referred to a broad range of nutrient and pharmaceuticals. They are known for various health benefits due to their pharmacological activity, nutritional values, dietary supplements for maintaining body health, and managing multiple metabolic processes and the regulation of normal body functions (Chan et al. 2021b; Chanda et al. 2019). The relation of food and its role in the treatment of multiple ailments has been established almost 25 decades ago by the father of modern medicine, Hippocrates, signified by the famous quote “Let food be thy medicine and medicine be thy food.” Various ancient civilizations such as Roman, Greek, and Egyptian have documented the use of herbal products, plants, and foods in treating and preventing diseases, which are now being evaluated as novel therapeutic agents by modern research (Helal et al. 2019). For example, nutraceuticals in the form of vitamins, minerals, dietary fibers, and polyunsaturated fatty acids, as well as flavonoids that are widely distributed in grapefruits, berries, onions, and green vegetables have been shown to reduce risks of developing cardiovascular diseases. Besides, plants that are rich in isoflavones, lycopene, and β-carotene possess antioxidative properties that can contribute to cancer-protective effect (Nasri et al. 2014). In terms of chronic respiratory diseases, curcumin, berberine, naringenin, green tea, gallic acid, and caffeine are examples of nutraceuticals that can be utilized to counter overproduction of reactive oxygen species as observed in chronic obstructive pulmonary disease (COPD) and lung cancer, whereas resveratrol, grape seed oil, coenzyme Q10, and lipoic acid exert remarkable anti-inflammatory activities that can be utilized to target various inflammatory pathways underlying the pathogenesis of asthma (Devkota et al. 2021a; Hardwick et al. 2021; Helal et al. 2019; Paudel et al. 2022b; Wadhwa et al. 2021). Similarly, medicinal plant such as *Alpinia galanga* (Ramanunny et al. 2022) and genus *Blepharis* (Dirar et al. 2021) possess significant anti-oxidant and anti-inflammatory activity that favors asthma management. Therefore, nutraceuticals have attracted medical researchers and they are widely regarded as the promising approach to existing therapeutic strategy for the effective management of different disorder, including asthma. The current review further elaborates the impact of the oxidative stress involved in the pathogenesis of asthma. Moreover, this review also focuses on the therapeutic potential of nutraceuticals as antioxidants in the asthma management.

## Oxygen radicals that mediate oxidative stress in asthma

A prominent trigger in the pathophysiological mechanism of oxidative stress is the imbalance due to excess generation of reactive oxygen species (ROS) (metabolized radicals of O2 and non-radical derivatives of O_2_ such as H_2_O_2_), endogenous nitric oxide (NO), reactive nitrogen species of NO (RNS), and protection against these radicals by the endogenous biological defense system (Kirkham and Rahman 2006). ROS and RNS are endogenously enhanced by various metabolic and energetic pathways of various cells together with the exposure to the environmental factors (e.g., air pollutants, pollen grains, metallic particles, and cigarette smoking) (Barnes 2006). These increased radicals drive the activation of various inflammatory and cell death pathways to initiate the detrimental events of the asthma like the epithelial cell damage and cell death, increased mucus production, increased release of danger-associated molecular patterns, eosinophils, and neutrophil infiltration into the airway lumen, and increased airway hyperresponsiveness and airway remodelling. The order of these reactive potentials of the free radicals and the oxidants are the superoxide anion (O_2_^−•^) which is considered the major precursor that is mainly produced by the oxidative phosphorylation from the mitochondrial source or from the reduced nicotinamide adenine phosphate (NADPH) oxidation catalyzed by NADPH oxidases (Nox) (Michaeloudes et al. 2022) followed by hydrogen peroxide (H_2_O_2_), hydroxyl radical (HO^•^), singlet oxygen (1O_2_), peroxyl radical (HO_2_^•^), nitric oxide (•NO), peroxynitrite (ONOO-), perhydroxy radical (HO_2_•), hydroperoxyl radical (ROOH^•^), hypochlorous acid (HClO), ozone (O_3_), and nitric dioxide (NO_2_) (Barnes 2006; Kirkham and Rahman 2006; Polosa 2002). TO mitigate the detrimental effects of these oxidative radicals, the pulmonary system has developed its own defense mechanism via activating different enzyme and non-enzyme network of antioxidants like superoxide dismutase (SOD), catalase (CAT), and glutathione-based enzymes such as glutathione peroxidase (GSH-Px), glutathione S transferase (GST), glutathione reductase (GSH), glutathione synthetase, NADPH oxidase, and Rho-oxidase (Imaoka et al. 2009).

### ROS-mediated oxidative stress in asthma

Enhanced ROS in the asthma pathogenesis is mainly contributed through the exposure to the environmental triggers including noxious gases, ozone, cigarette smoke, and also by activation of various inflammatory and phagocytic cells including eosinophils, monocytes, macrophages, and neutrophils when stimulated by the exogenous irritants (Henricks and Nijkamp 2001). The released ROS including superoxide ion (O_2_^−^) and radical hydroxylic ion (OH) due to their high instability with the presence of unpaired electrons that have the potential to initiate oxidation by several cellular substances including the lipids, proteins, and DNR to initiate the disruption of normal cell functions (Imaoka et al. 2009).

**Fig. 1:**
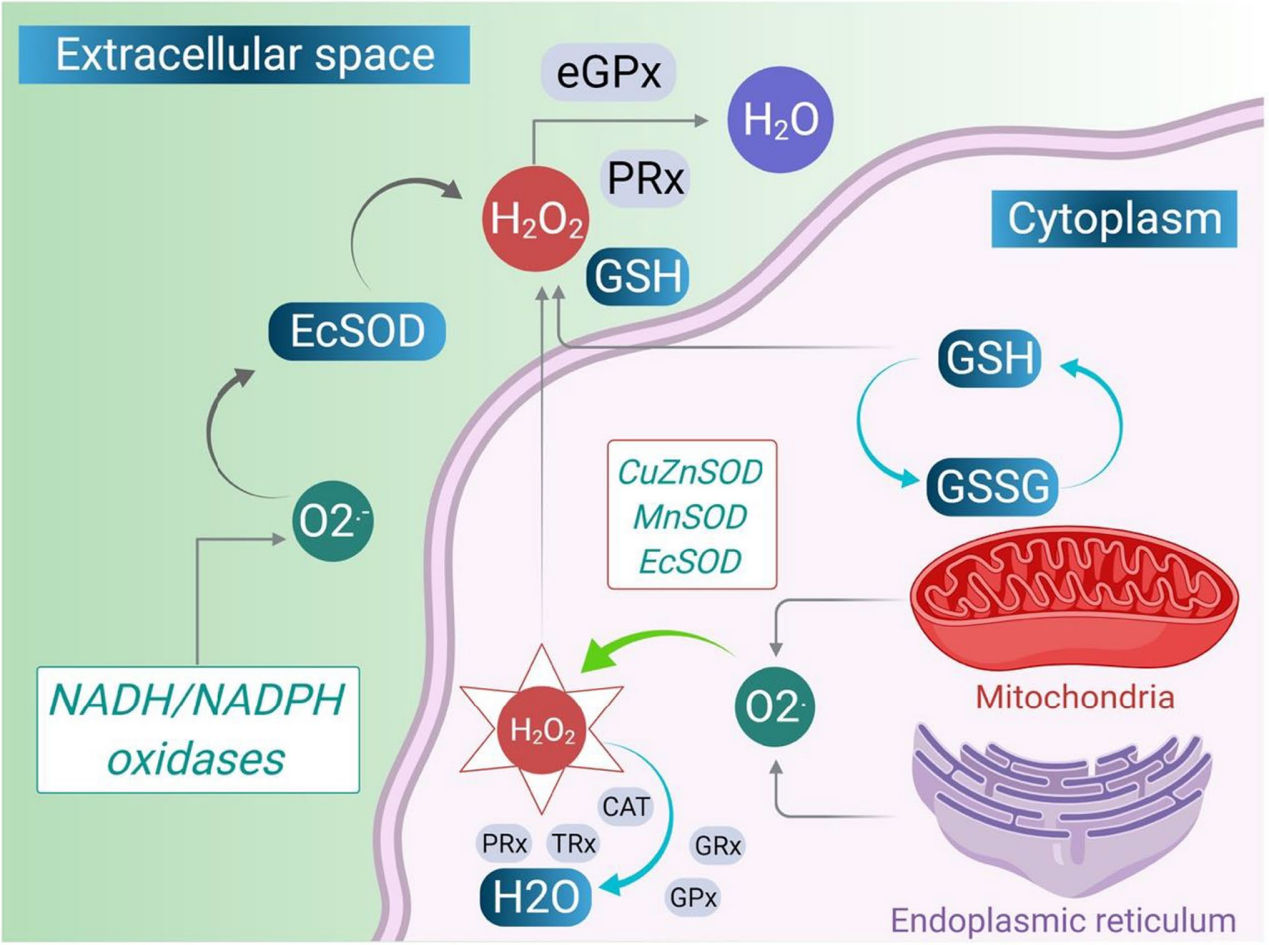
Intracellular and extracellular ROS generating systems. The macrophages and eosinophils generate free O_2_^−^. These O_2_^−^ are converted into water when they interact with superoxide dismutase (SOD) (dismutation). When ferric ions act on O_2_^−^, they get reduced to iron (Haber-Weiss chemistry), and they release free OH, which is the most reactive and harmful substances of ROS. H_2_O_2_ is also formed by the interaction with iron ion (Fenton chemistry)(Polosa 2002). Another pathway of producing OH is through the oxidation of hypochlorous acid (HOCl). HOCl is produced by neutrophils. They contain a high concentration of myeloperoxidase (MPO). MPO chlorinates H_2_O_2_ (created by O_2_ dismutation) to produce HOCl. HOCl is also a powerful oxidant that can cause several harms. Eosinophil epoxidase (EPO) is a protein found in both eosinophils and neutrophils. EPO brominates H_2_O_2_ to form HOBr, which is also a reactive species, in the same way that MPO does. The H_2_O_2_, which is less reactive, is converted to water by catalase or by glutathione peroxidase (Fig. 1). Through the electron donation to the biological system, ROS (OH, HOCl, HOBr) are highly unstable and interact with a wide range of molecules, leading to lipid peroxidation dysfunction (LPO) and increased co-inflammatory signaling. All of these cause modifications in cellular functions of the inflamed lung

**Fig. 2:**
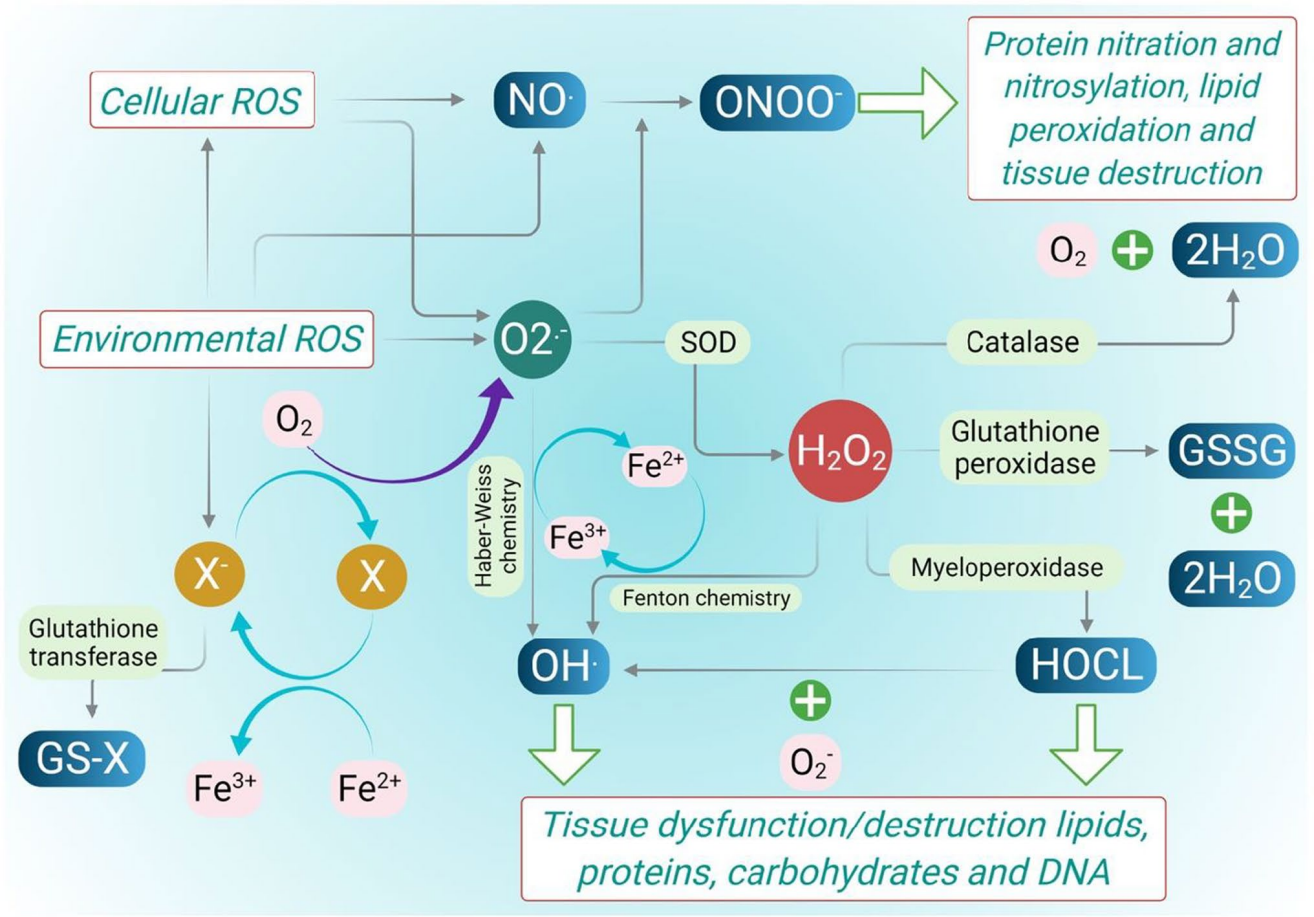
Molecular pathways of ROS and RNS generation. Degradation of arachidonate-based phospholipids produces other bioactive molecules such as 1-palmitoyl-2-(5) oxovaleroyl-sn-glycero-3-phosphorylcholine (POVPC), and 1-polmitoyl-2-epoxyisoprostane-sn-glycero-3-phosphorylcholine (PEIPC) and 1-palmitoyl-2-glutarouyl-sn-glycero-3-phosphorylcholine (PGPC). These are all proinflammatory molecules which increases infiltration of monocytes, neutrophils, and its associated cytokines. Several clinical studies have documented the destructive characteristics of ROS. Exhaled H_2_O_2_, high isoprostane levels in urine, bronchoalveolar lavage fluid (BALF), increased levels of eosinophils in blood and BALF, and increased neutrophil-derived MPO in blood are all increased in asthmatic patients as a result of ROS generation. The figure was adapted from Polosa (2002) and was reproduced

When ROS are produced in close proximity to the cell membrane, oxidation of membrane phospholipids occurs, resulting in the formation of lipid peroxidation molecules (LPOs) and the generation of several lipid hydroperoxidase molecules in the cell membrane. LPOs generate reactive aldehydrogen formation and other bioactive molecules (Fig. 1) (Rahman et al. 2006). This reactive aldehydes like acrolein and 4-hydroxy-2-nonnonenal (4-HNE) are highly diffusible and cause apoptosis to activate various cellular-mediated pathways. They also form adducts with histidine, cystine, and lysine residues, such as histodeacetylase (HADC-2) and also react with collagen and fibrinonectin. Cumulating all these events finally leads to changes in the cellular function that lead to progression of the asthma disease (Fig. 2).

### RNS-mediated nitrosative stress in asthma

**Fig. 3:**
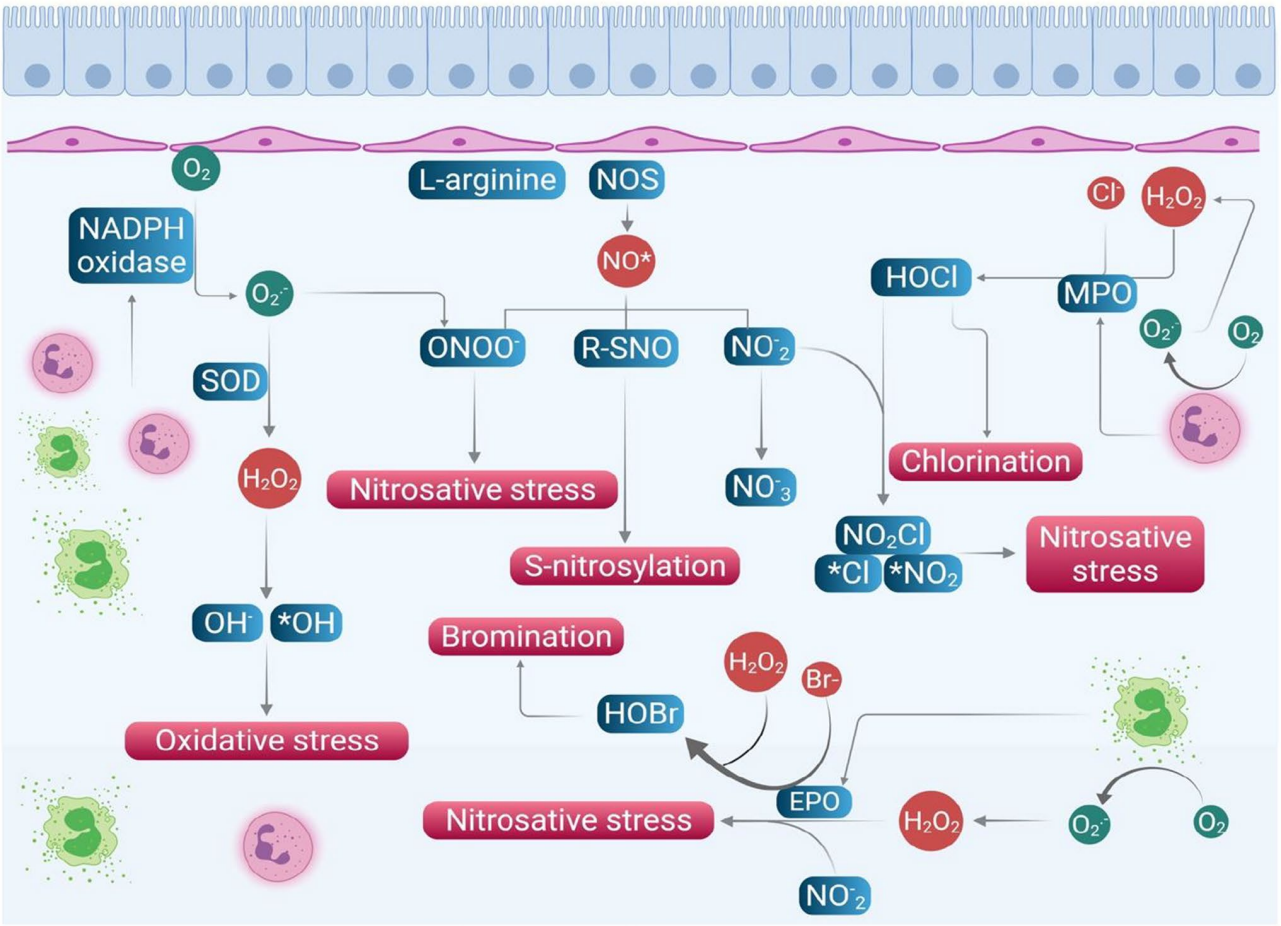
Components involved in RNS metabolism. Mutliple elements are involved in the RNS metabolism of which NO is the major component which is increased in the presence of oxidative stress. The increased NO reacts with oxygen to produce various nitrogen reactive radicals to induce the nitrosative stress which cause cell damage, mitochondrial dysfunction, and increased AHR. (NOS, nitric oxide synthase; MPO, myeloperoxidase; EPO, eosinophil peroxidase; SOD, superoxide dismutase). The figure was adapted from Rahman et al. (2006)

Nitric oxide (NO) and NO-derived reactive nitrogen species (RNS) are another category of the free radicals which triggers the initiation of nitrosative stress in modulating the airway function in asthma pathogenesis (Zuo et al. 2014). The generation of these RNS endogenously is indirectly linked with the oxidative stress and the ROS. NO is produced by respiratory epithelium and inflammatory cells like neutrophils, macrophages, airway nerves, and endothelium and plays a key role in mediating the vascular tone and bronchotome of airway smooth muscle cells (Vasconcelos et al. 2021; Zuo et al. 2014). NO is produced by the various nitiric oxide synthases (NOS) as a by-product during the metabolism of the L-arginine and also during the interaction of L-citrulline with oxygen using NADPH-dependent mechanism (Vasconcelos et al. 2021). Nitric oxide synthase (NOS) has three isoforms: constitutive neural NOS (nNOS), inducible NOS (iNOS), and constitutive endothelial NOS (eNOS) (Andrew and Mayer 1999). However, there are only two forms of functional NOS in the airways which are constitutive NOS (cNOS) and inducible NOS (iNOS) (Ricciardolo 2003). The cNOS produces fento or picomolar concentration of NO which is physiologically important in involving in various regulatory mechanisms including bronchodilation, bronchoprotection, and anti-inflammatory action via interactions with guanyl cyclase (production of cGMP) and sulfhydryl groups (production of s-nitrothiols)(Zuo et al. 2014). In contrast, iNOS is induced in the presence of proinflammatory cytokines (TNF α, IFN-g, IL-1β) by the stimulation of several transcription factors. iNOS releases nanomolar concentration of proinflammatory NO several hours after exposure and continues to sustain for hours or days. NO has one unpaired electron and thus readily interacts with oxygen or transition metals to form bioactive reactive nitrogen species (Fig. 3). The reaction of NO and O_2_ produces peroxynitrite ions (ONOO-), a highly reactive oxidant species that interacts with tyrosine to produce the stable product nitro tyrosine (R-SNO), which is known as S-nitro(syl)ation. NO_2_ is formed from NO, which is also reactive upon combining with HOCl (produced by MPO as described above) or with HOBr (from EPO) to produce nitrosyl chloride (NO_2_Cl) or nitrosyl bromide (NO_2_Br) which degrade into nitrite and Cl^−^ or Br^−^ (Fig. 3). The increased RNS are highly reactive molecules which cause the nitrosative stress and react with various biomolecules of the cells and tissue of the airways and trigger a cascade of different mechanisms which causes cell damage, DNA damage, and mitochondrial and protein dysfunction to induce airway damage and airway hyperresponsiveness (Rahman et al. 2006).

## Mitochondria-associated oxidative stress in asthma pathogenesis

As previously mentioned, endogenous oxidative stress and the formation of various reactive radicals are mainly generated either due to mitochondrial metabolism dysfunction or due to increased levels of the oxidant-mediated enzymes that are involved in the oxygen catalyzation. Mitochondria are the cytoplasmic organelles which possess various cellular and biological functions of which bioenergetics is a primary function for the production of energy in the form of adenosine triphosphate (ATP). Despite the involvement in bioenergetics, mitochondria are also involved in maintaining the various cellular homeostasis functions including calcium regulation, alteration of redox potential, and release of protein that stimulates the caspase family of proteases (Mabalirajan and Ghosh 2013; Reddy 2011). Mitochondria can also sense the exogenous upstream triggers such as inflammation, tobacco, smoke, infection, and environmental insults and in turn can respond to such stimuli via changed mitochondrial protein expression and structure. Equally, mitochondrial dysfunction has downstream effects on cytosolic and mitochondrial airway contractility, proliferation, gene and protein housekeeping, calcium regulation, fibrosis, responses to oxidative stress, apoptosis, and metabolism (Chellappan et al. 2021; Prakash et al. 2017).

**Fig. 4:**
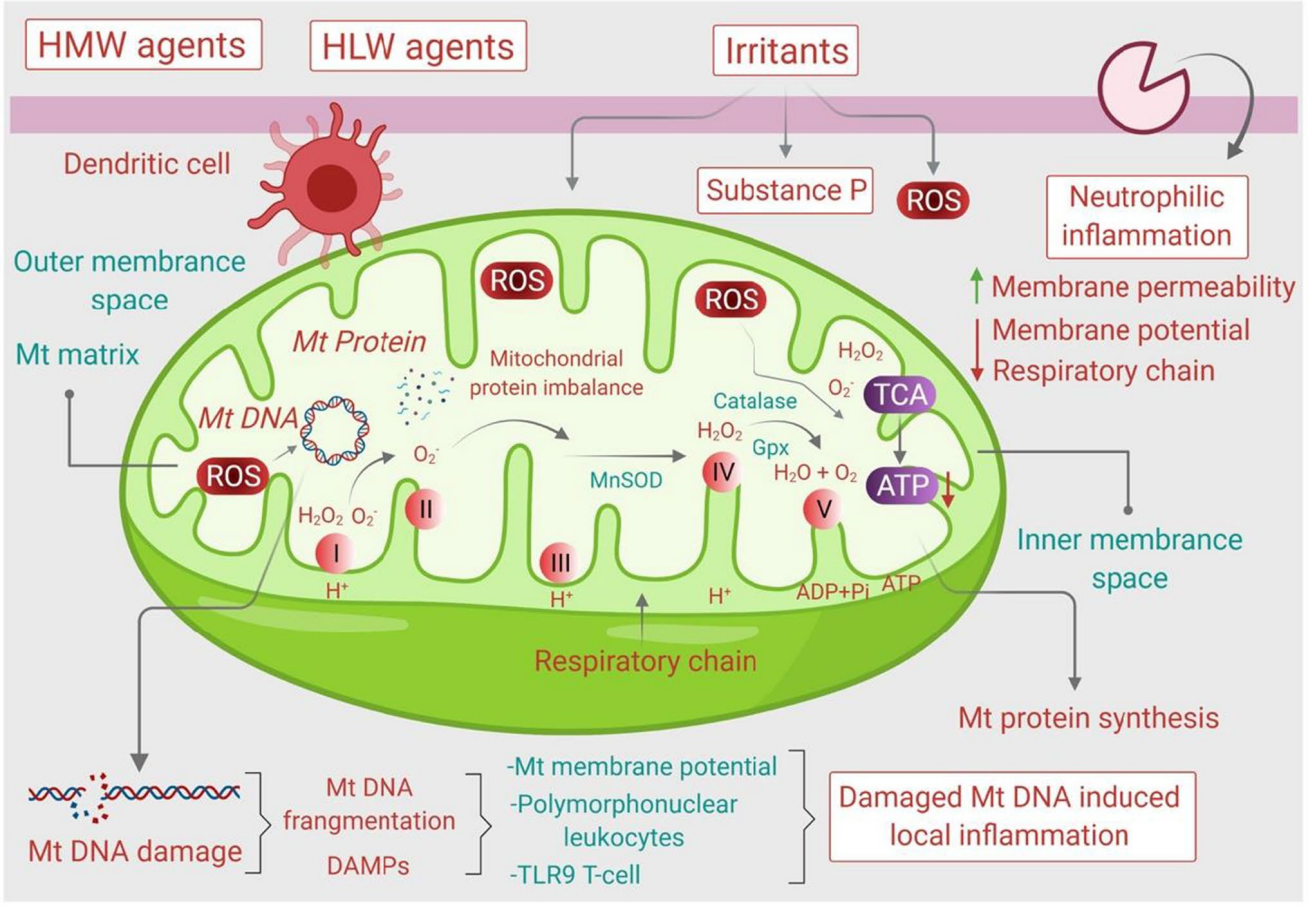
Mitochondrial oxidative stress in asthma. Mitochondria in the presence of various environmental stressors alter its energetic process to release various mitochondrial-derived danger molecules including mitoROS and mitoDNA to cause mitochondrial damage. The associated mitochondrial damage releases the danger-associated factors into the lung environment to induce the local inflammation and AHR

Mitochondria are not only involved in the bioenergetics and bio-maintenance of various cellular functions but also involved in the modulating the innate immune system via controlling the ROS and RNS generation. Damaged and dysregulated mitochondria lead to decreased formation of ATP and increased endoplasmic reticulum (ER) stress. Mitochondrial oxidative stress and ER stress further lead to cell apoptosis. Mitochondrial dysfunction plays a crucial role in the bioenergetics metabolism and non-energetics pathogenesis in several pulmonary diseases and the lack of mitochondrial homeostasis leads to cell injury and cell death. ROS activated by oxidative stress and inflammatory antigens are the major factors for increasing mitochondrial DNA (mtDNA) damage, dysregulation of the tricarboxylic acid (TCA) cycle, and dysregulation of the electron transport chain (Reddy 2011). Furthermore, increased level of interleukin (IL-4) is another potential cause of mitochondrial dysfunction in asthmatic patients. It also induces 12/15-lipoxygenase (12/15-LOX) which is the key cause of asthma. IL-5, IL-13, and ovalbumin (OVA) specific IgE and airway hyperresponsiveness (AHR) are also responsible for mitochondrial dysfunction (Mabalirajan et al. 2009) and thus conclude that Th2-dominant response enhances mitochondrial oxidative stress in airways (Mabalirajan et al. 2008). The molecular mechanisms on how the mitochondrial oxidative stress influenced the pathogenesis of asthma is represented in Fig. 4.

### Nutraceuticals that target mitochondrial stress in asthma

Various nutraceuticals have been identified which have shown positive effects against mitochondrial dysfunction for the treatment of asthma (Chan et al. 2021b). In one of the studies, Mabalirajan et al. reported the effects of vitamin E against mitochondrial dysfunction for the treatment of asthma. In order to evaluate various pathological pathways, in vitro studies were conducted in male BALB/c mice. The results revealed that vitamin E attenuated the production of IL-4, IL-5, IL-13, OVA-specific IgE, and AHR. Vitamin E also reduced allergic skin sensitization and airway inflammation (Mabalirajan et al. 2009). Gheware et al. reported the effects of *Adhatoda vasica* extract against hypoxia-induced mitochondrial dysfunction in acute allergic asthmatic mice. The results indicated that oral administration of *Adhatoda vasica* extract significantly attenuated IL-17A and hypoxia-inducible factor-1α (HIF-1α). The study showed that *Adhatoda vasica* extract produced anti-inflammatory effects and acted as an inflammatory marker which is responsible for mitochondrial dysfunctions (Gheware et al. 2021). Zheng et al. have studied the effects of curcumin against ER stress and mitochondrial dysfunction. Curcumin downregulated the level of NF-κB signaling pathway through its anti-inflammatory and immunomodulatory properties. It has also been observed to attenuate the level of CD4+ T cells and further downregulated ER stress and mitochondrial dysfunctions (Zheng et al. 2013). Lee et al. investigated the effects of resveratrol and its anti-inflammatory and anti-asthmatic activities in experimental mice model. Resveratrol significantly attenuated the level of Th2 cytokines such as IL-4 and IL-5 in plasma and bronchoalveolar lavage fluid and also effectively suppressed airway hyperresponsiveness and mucus hypersecretion, in the asthmatic mouse model (Lee et al. 2009).

Fiorani and co-workers had investigated the effect of quercetin against mitochondrial damage in Jurkat cells. Quercetin has been reported to prevent ROS such as peroxynitrite (ONOO−) or attenuation of extracellular oxidants. It has also attenuated the higher level of lipid peroxidation induced by ONOO−. Quercetin prevented mitochondrial damage and had caused its redistribution to the cytosol by stimulation of plasma membrane oxidoreductases (Fiorani et al. 2010). Liang et al. have investigated the role of thymoquinone against ultraviolet A irradiation–induced damage on skin keratinocytes and accumulation of ROS on human keratinocyte cell culture (HaCaT). In this study thymoquinone significantly improved ultraviolet A irradiation–induced cytotoxicity in HaCaT cells. It has also shown improved mitochondrial function in HaCaT cells which attenuated cellular apoptosis. Thymoquinone activated NrF2/ARE pathways and inhibition of the COX-2 inflammatory mediator (Liang et al. 2021).

Lee et al. studied the effects of glycyrrhizin against 3-morpholinosydnonime induced cell death and mitochondrial dysfunction in lung epithelial cells. 3-morpholinosydnonime causes several cellular dysregulations such as nuclear damage, cytosolic accumulation of cytochrome c, reduction in the mitochondrial transmembrane potential, activation of caspase-3, enhancement in the formation of ROS, and depletion of GSH. Glycyrrhizin was observed to attenuate the level of 3-morpholinosydnonime and thereby overcame the mitochondrial damage, accumulation of ROS, and GSH depletion caused by 3-morpholinosydnonime in lung epithelial cells. Glycyrrhizin further minimized the mitochondrial permeability transition in lung epithelial cells that led to the activation of caspase-3 and release of cytochrome c that caused the depletion of 3-morpholinosydnonime (Lee et al. 2007).

## Non-mitochondrial stress–associated oxidative stress in asthma

### NADPH oxidase

The important non-mitochondrial enzymes which are required for the production of superoxide radicals from free oxygen are the NADPH oxidases that produce superoxide radical, a major ROS involved in the asthma pathogenesis (Segal et al. 1981). NADPH oxidases that belong to the NOX family are the key ROS producing enzymes that mainly activate in the phagocytic cells like macrophages and neutrophiles to create an oxidative burst in the rapid elimination of the invading microorganisms (Panday et al. 2015). The critical role of NADPH oxidase as a transmembrane protein involves in transferring electrons from NADPH to FAD to reduce the oxygen for the generation of the superoxide radical, and the dysfunction of these oxidases leads to excess ROS production to activate various cellular and inflammatory pathways that are involved in the progression of various diseases (Snezhkina et al. 2019). To date, seven different homologues of the NADPH enzymes have been identified which consist of NOX1 to NOX 5 and the two NOX-5 like dual oxidases including DOUX1 and DOUX2 (Lambeth 2004).

NADPH oxidase consists of five protein components that render its structure complex. Among these two components are membrane bound (P22^PHOX^ and GP91^PHOX^ and G-protein Rap1A), while three components are present in the cytosol in a resting phase (P40^PHOX^, P47^PHOX^, and P67^PHOX^). The bonding proteins are also present in cytosol with the other components: rac1 or rac2, the two small molecular weight guanine nucleotide proteins. When cytosolic parts migrate to the cell membrane, NADPH oxidase is activated. The family NOX is gp91PHOX homologous and forms p22PHOX heterodimer except NOX5 and DOUX. As an additional EF intracellular Ca2+ binding hand domain, both NOX5 and DOUX1/DOUX2 are calcium-sensitive, whereas NOX1 to NOX3 are not directly calcium-dependent for activation (Han and Lee 2000). NOX4 requires p22PHOX but requires no other co-factor such as NOX1 to NOX3. GP91PHOX is one of the five components that supports electron transfer through NADPH oxidase (NOX2 is also known as GP91PHOX). P67PHOX is an auxiliary protein which is required for oxidase activation. During stimulation of the cell, they move from cytosol to membrane following the heavy phosphorylation of p47PHOX (Babior 1999). Simultaneously, P22PHOX and gp91PHOX assemble on membranes with Rap1A, p40PHOX, p47PHOX, and rac1 or rac2 (Babior et al. 2002). This entire assembly activates the oxidase, allowing it to transfer electrons from the substrate to oxygen. When neutrophils lack p47PHOX and p67PHOX, they are unable to produce superoxide, resulting in chronic granulomatous disease, which is a genetic disorder (Sheppard et al. 2005).

### Role of NADPH oxidase in asthma and generation of ROS

**Fig. 5:**
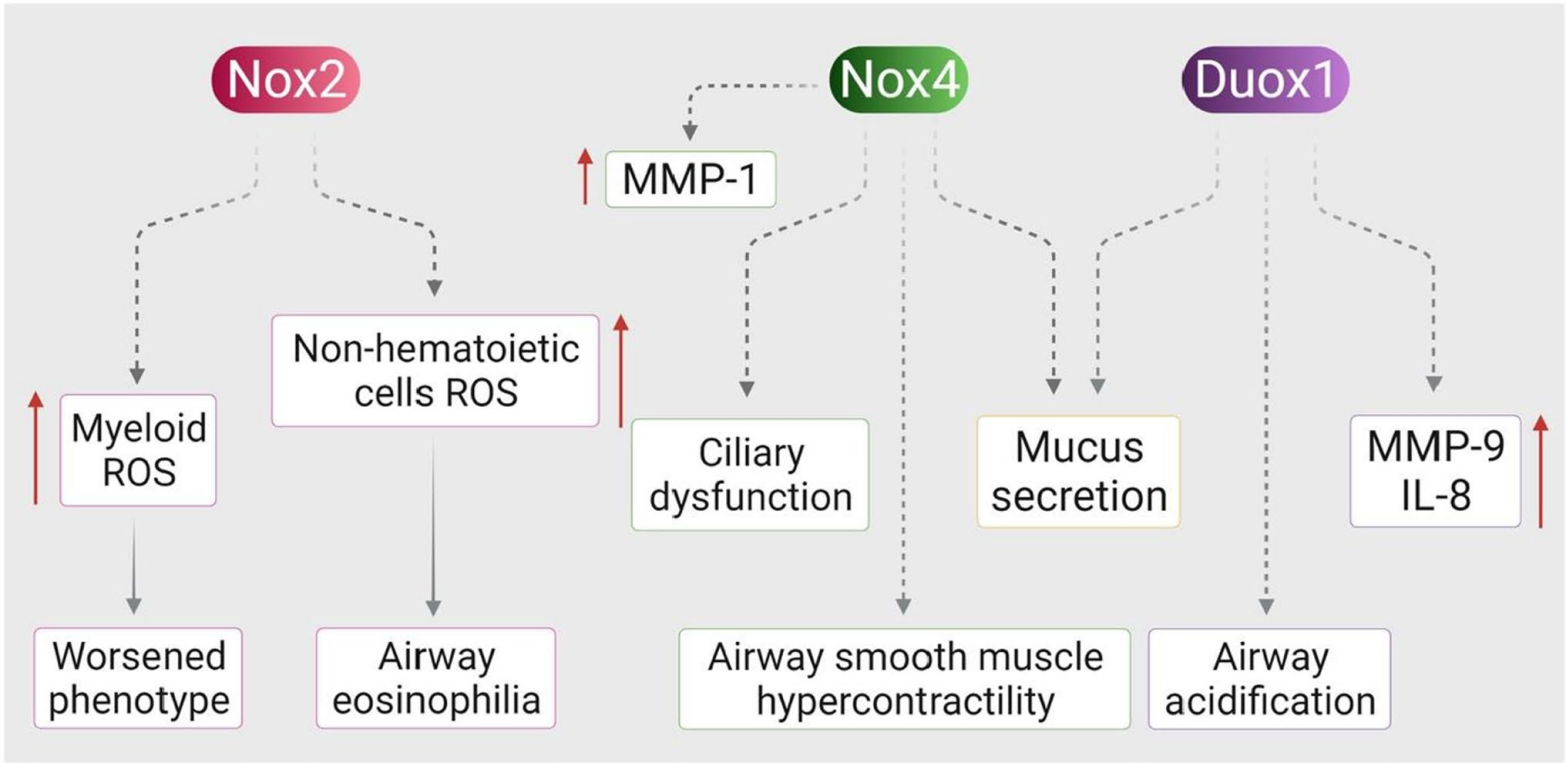
Role of NOX in asthma. NOX2, NOX4, and dual oxidase 1 (Duox1) play critical roles in asthma through various mechanisms. Both Duox1 and Nox4 stimulate mucus secretion and matrix metalloprotease (MMP) production in airway epithelial cells. In addition, Duox1 enhances airway acidification and Nox4 induces ciliary dysfunction and airway smooth muscle hypercontractility. Nox2 expressed in myeloid and non-hematoietic cells plays distinctive roles in asthma. Nox2 expressed in myeloid cells is believed to mediate the worsened phenotype of asthma, while Nox2 expressed in the lung structure cells mediates airway eosinophilia. The figure was adapted from Harijith et al. (2017) and was reproduced

The main non-mitocndrial source of ROS generation via superoxide is NADPH oxidase (Fig. 5). ROS is a beneficial inflammatory process that encloses harmful bacteria and retains the body’s innate immunity (Lee and Yang 2012). However, over-production of ROS gives rise to inflammatory diseases of the respiratory system such as acute respiratory distress syndrome, asthma, cystic fibrosis (CF), COPD, tissue destruction, and remodelling (Harijith et al. 2017). Superoxide anion is the primary product of NOX/DOUX dismutation to hydrogen peroxide. However, DOUX1/2 and NOX4 generate H_2_O_2_ without the use of a superoxide intermediate (Lee et al. 2006). NOX2 is present in neutrophils and eosinophils whereas ROS is the primary source of allergic inflammation. These myeloid cells generate ROS and contribute towards the exacerbation of asthma (Fahy 2009). NOX4 has the ability to detect oxygen, cells, apoptosis, fibrosis, and inflammation. They are found in epithelial cells, smooth muscle cells, and lower airway mesenchymal cells. In pharmacological inhibition and genetic approaches of asthma model, increased smooth muscle contractility in the airway was repudiated in NOX4 (Clempus et al. 2007). NOX4 and DOUX1 levels in the neutrophilic murine model of asthma are elevated, whereas only DOUX2 is elevated in the non-neutrophilic murine model of asthma.

Pharmacological NOX4 antagonist was reported to improve ciliary function in a neutrophilic asthmatic murine model. Among all NOX-proteins, external hydrogen peroxide, which promotes oxidative stress and leads to matrix metalloprotease (MMP)-1, can be increased in normal human nasal epithelial cells (Waghray et al. 2005). MMPs are also released by other airway cells such as interstitial cells, vascular smooth muscle cells, and infiltrated inflammatory cells such as macrophages; thus, MMPs can be a crucial target to mitigate chronic respiratory disease (Mehta et al. 2021a). The main source of hydrogen peroxide is DOUX1/2 in the upper respiratory system. Immuno-histochemical analysis has identified the presence of DOUX1 at apical surface of tracheobronchial tree and epithelial cells of alveoli while DOUX2 was found in salivary and submucosal glands (Geiszt et al. 2003). In allergic asthma, activation and expression of epidermal growth factor receptor (EGFR) signaling are signs of mucous metaplasia and airway remodelling. It has been discovered that genetically inhibiting DOUX1 reduces EGFR and reverses the signs and symptoms of asthma in a murine model. In allergic asthma, DOUX1 secretes mucous via TNF-alpha-converting enzymes and increases the levels of several inflammatory mediators such as IL-8 and matrix metalloprotease-9. DOUX1’s innate host defense property causes it to secrete H+ and increase airway acidification (Tyner et al. 2006). DOUX2 is involved in cell motility and wound healing. Although the role of NOX1 in asthma is unknown, NOX1 plays an important role in alveolar cell injury during hyperoxia in knockout mice (Carnesecchi et al. 2009).

### Rho GTPase

Rho GTPase is a subfamily of the Ras superfamily that binds to GTP and performs many cellular functions such as cell growth and development, cell regulation, cell motility, transcription regulation, and actin cytoskeleton rearrangement (Van Aelst and D’Souza-Schorey 1997). RHO GTPase in animals contains approximately 20 proteins. Rho (A, B, C), Rac (Barnes 2006; Kirkham and Rahman 2006; Polosa 2002), Cdc42, TC10, TCL, Chp (Barnes 2006; Kirkham and Rahman 2006), RhoG; Rnd (Dozor 2010; Kirkham and Rahman 2006), RhoBTB (Abo et al. 1991; Kirkham and Rahman 2006; Polosa 2002), RhoD, Rif, and TTF are all members of the Rho family (Etienne-Manneville and Hall 2002). Rho GTPase is activated in the cytosol by guanine nucleotide exchange factors (GEFS), which convert Rho from GDP to GTP (from inactive to active) (Hanna and El-Sibai 2013). GTPase activating proteins (GAPs) inactivate Rac. RhoGDI keeps Rac inactive in the cytosol, but it has also been discovered that Arghap1 and Arghap25 inactivate Rac2 in leukocytes (Lőrincz et al. 2014). Researchers have identified approximately 30 Rac GEFs in animals, including VAV1, VAV2, and Tiam 1, which can stimulate ROS production via NADPH (Hanna and El-Sibai 2013).

### Role of Rho GTPase in activation of NADPH

Under normal circumstances, NADPH oxidase activates Rac. NOX1 and NOX2 are in turn activated by Rac. However, the activation of NOX3 is unclear, although researchers have identified the Rac binding site in NOX3. No binding or activation of Rac has been reported in NOX4 (Quinn et al. 1995). RACs are essential for the activation of NOX1 and NOX2 in signal transmission for growth factors, cytokines, cell-cell proliferation, chemotaxis, and phagocytosis (Quinn et al. 1995). The roles of NOX1 and NOX2 oxidase activation via Rac1 and Rac2 in cells differ; for example, in monocytes, Rac1 is the primary activator, whereas in neutrophil progenitors, Rac2 is important. In mice, genetically inhibiting RAC1 in leukocyte cells has been reported to reduce actin assembly and chemotactic factor without affecting ROS generation (Hordijk 2006).

Rho GTPase is found in the cytosol alongside p40PHOX, p47PHOX, and p67PHOX. Activation of NOX2 in neutrophils during phagocytosis requires the small GTPase for the production of superoxide, which generates ROS to engulf the microbes. External stimuli activate the cell, and Rho GTPase is activated via GEFs, which convert GDP to GTP and RAC GTP binding, which is directly attached to P67PHOX in the cytosol. This binding interacts with NOX2 via the membrane’s active domain (AD) to produce superoxide (Abo et al. 1991). NOX1 is the first animal oxidase enzyme found in non-phagocytic cells, such as colon epithelial cells and smooth vascular muscle cells (Knaus et al. 1991). NOX1 activation requires soluble proteins such as Nox activator 1 (Noxa1) and Nox organizer 1 (Noxo1), as well as the GTP binding Rac, which has the ability to directly bind with Noxa 1 and easily generates superoxide through NOX1. NOX1 is capable of producing a large amount of superoxide in the absence of any cellular stimuli (Mizuno et al. 1992).

### Role of Rho GTPase in asthma

**Fig. 6:**
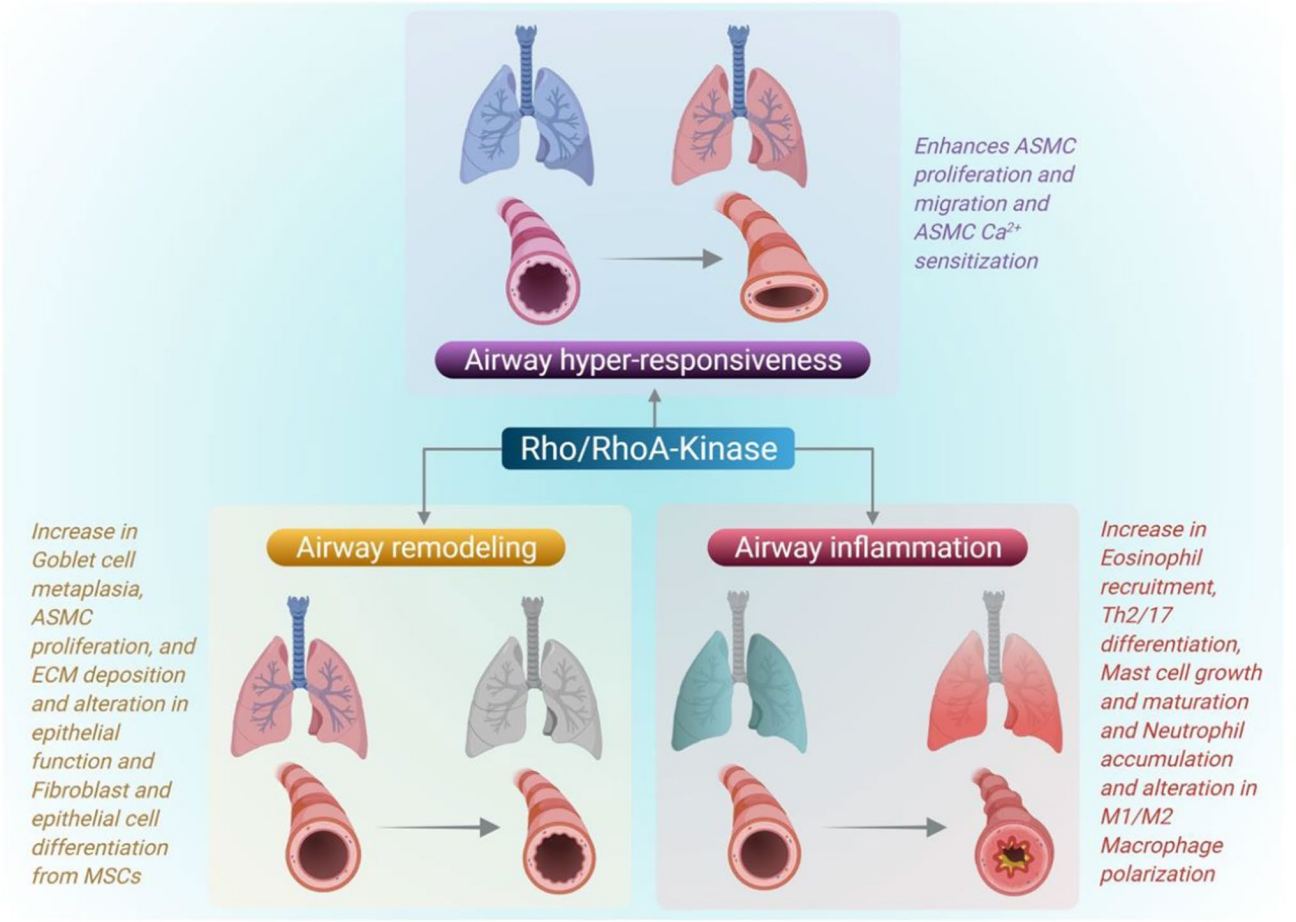
Role of Rho GTPase in the regulation of asthma. Rho GTPase mainly RhoA-kinase activates the Rho-activated kinase (ROCK) to induce the airway smooth muscle proliferation to induce AHR. Also, RhoA kinase also involved in eosinophil recruitment, mast cell activation, and altered macrophage polarization which play a key role in the pathogenesis of asthma. The figure was adapted from McCarty et al. (2021) and was reproduced

Rho family of GTPases are the fundamental enzymes that orchestrate various cellular homeostasis functions including cell division, cell cycle progression, and actin cytoskeleton assembly (Phuyal and Farhan 2019). Rac, Rho A, and Cdc42 are the main Rho family regulators required for lamellipodia, filopodia, and actin polymerization (Srinivasan et al. 2003). Autocoids activate G-protein-coupled receptors on smooth muscle, as well as RhoA and phospholipase C, which depolarize the plasma membrane and induce calcium channels. Rho A is activated by GEFs, which binds Rho A to GTP (Shimokawa et al. 2016). RhoA activated Rho-activated kinase (ROCK), which is an antagonist of myosin light chain phosphatase (MLCP), relaxes the smooth muscle. CPI-17 protein complex activated via protein kinase C also inhibits MLCP by increasing MLC phosphorylation. IP3 and DAG are stimulated by phospholipase C, which aids in the influx of calcium ions and the increase of calcium through the sarcoplasmic reticulum. Increased calcium levels in the cell activates myosin-light-chain-kinase (MLCK) via calmodulin (CaM) (Snetkov et al. 2001). The NOX4 isoform of NADPH oxidase increases RhoA activity in the smooth muscle cells (McCarty et al. 2021). The role of RhoA in the regulation of asthma is summarized in Fig. 6.

## Nutraceuticals that target non-mitochondrial stress

In preventing and treating various respiratory disorders, nutraceuticals play an important role. Natural substances and nutritional supplements are becoming ever more widely recognized in preventive health care today. Globally, the promotion of functional foods, drinks, and herbal supplements meets the nutritional and health needs of each person. Asthma control may be clinically assistive to nutraceutical substances that control lung oxidative stress (PhyCB, NAC, LA, or ferulic acid, selenium, and zinc), promote NO (citrulline, high dose folates, and H_2_S (NAC, taurine), and directly cause bronchodilatation via calcium modulation (glycines, mg). These micro- and innervated enzymes contribute to modulating the immune system and reduces the risk of various conditions in humans, including respiratory conditions (McCarty et al. 2021; Qu et al. 2017).

### Phytochemicals targeting non-mitochondrial oxidative stress

There are several sources of non-mitochondrial stress such as plasma F2-isoprostane, enzymes such as myeloperoxidase, eosinophil peroxidase (EPO) (Panth et al. 2016b), lipid peroxidation product (such as isoprostanes, lipid hydroperoxides, oxidized low density lipoprotein) (Tarafdar and Pula 2018), and nitric oxide (NO) that are produced via inducible nitric oxide synthase pathway (Paudel et al. 2016). Immune cells such as eosinophils are major source of NO-derived oxidants (such as 3-nirotyrosine), and they generate ROS through EPO catalyzed oxidation (MacPherson et al. 2001). Peroxidase and NAPDH oxidase can oxidize numerous substrates to produce reactive oxygen species (ROS) and therefore are the potential targets of antioxidant molecules (Mathur and Vyas 2013; Schaffer and Bronnikova 2012). Several nutraceuticals are explored for their promising antioxidant activity both in vitro and in vivo (Chan et al. 2021b; Manandhar et al. 2018; Panth et al. 2016a; Paudel and Panth 2015; Prasher et al. 2020). Paudel et al. (2020a) evaluated the antioxidative potential of rutin-loaded liquid crystalline nanoparticles in lipopolysaccharide-induced oxidative stress in human bronchial epithelial cells (BEAS-2B) in vitro. It was observed that the rutin formulation showed potent antioxidant activity by inhibiting the total cellular ROS and NO at a dose of 5 μM (Paudel et al. 2020b). The antioxidant activity was further validated by gene expression and was found that rutin inhibited specific genes (NADPH oxidase *(Nox)-4*; *Nox2B*) and upregulated the antioxidant genes NADPH quinine oxidoreductase-1 (*NQO1*) and γ-glutamyl cysteine synthetase catalytic subunit (*GCLC*) (Mehta et al. 2021c; Vyas et al. 2017). Baicalin (a flavonoid) and liensinine (an alkaloid) inhibit NO production by reducing the protein expression of iNOS and inhibit serum lipid peroxidation to show anti-oxidant activity (Jun et al. 2021; Paudel et al. 2020b). Similarly, in vivo mice model of asthma has also shown that nutraceuticals (plant extract or single compound) target various non-mitochondrial stress. *Eriobotrya japonica* leaf extract at a dose of 100 and 200mg/kg body weight was found to inhibit the EPO and NO in BALB/c mice sensitized and challenged with ovalbumin (Kim et al. 2020). Another mice model revealed that a nutraceutical supplement of apocynin, lipoic acid, and probiotics has a positive influence on the antioxidant enzyme in obese asthmatic mice. The lung tissue of mice administered with supplements separately for 12 weeks showed an increase in the activity of superoxide dismutase (SOD) in apocynin-treated group. Similar increases were observed with glutathione reductase activity in the lipoic acid–treated group and glutathione peroxidase activity in probiotics-treated group (Kleniewska and Pawliczak 2019). Sakuranetin (a flavonoid) treatment reduced pulmonary oxidative stress by inhibiting the 8-iso-prostaglandin F2a in lung tissue of ovalbumin-induced mice model of asthma (Sakoda et al. 2016). Astragalin is another flavonoid that protects the tissues from LPS-induced epithelial cell apoptosis and etoaxin-1 induction by targeting oxidative stress-response mediated through mitogen-activated protein kinase (MAPK) signaling. This study was performed in vitro in BEAS-2B cells where astragalin at a dose of < 20 mM inhibited intracellular total ROS production and protein expression of PLCg1, PKCb2, and NADPH oxidase subunits of p22^phox^ and p47^phox^. The phosphorylation of MAPK family protein JNK and P38 was also inhibited by astragalin proving its potent antioxidant activity in asthma in vitro (Cho et al. 2014).

Similar mechanism was also observed with morin (a flavonoid from Moraceae plant) treatment to human bronchoepithelial cells where morin modulated oxidative stress responsive-MAPK pathway. The antioxidant activity of morin was shown by significant decrease in total cellular ROS and decreased phosphorylation of MAPK family protein (ERK, JNK, P38) (Ma et al. 2016). Resveratrol is a well know polyphenol with antioxidant potential. A mice model of obesity (induced by high-fat diet [HFD])-associated allergic airway inflammation (induced by ovalbumin) was established to investigate the potential of resveratrol to reverse oxidative damage by measuring the total ROS, enzyme, and protein expression. It was revealed that resveratrol significantly inhibited p47phox and iNOS protein expressions, ROS production, and elevated the SOD levels in lung tissues in mice treated with HDF and ovalbumin compared to control mice (André et al. 2016). Collectively, these in vitro and in vivo studies highlight various promising nutraceuticals with antioxidant potential in the management of asthma. Further in-depth mechanism–based translational research is essential to validate them as therapeutic alternative (Table 1).

**Table 1:** Nutraceuticals targeting non-mitochondrial stress in vitro and in vivo

Nutraceuticals	Study design	Findings	Reference
Rutin-LCNs	In vitro study on BEAS-2B	Inhibit total cellular ROS	(Paudel et al. 2020b) (Mehta et al. 2021c)
		Inhibits gene expression of *Nox*, *NOX2B* and upregulate *NQO-1* and *GCLC*	
Baicalin	In vitro (macrophage) and serum lipid peroxidation assay	Inhibits NO via iNOS pathway, inhibits serum lipid peroxidation	(Paudel and Kim 2020)
*Eriobotrya japonica*	Ovalbumin induced mice model of asthma	Inhibits EPO and NO in BALF of BALB/c mice and serum lipid peroxidation	(Kim et al. 2020)
Apocynin	Ovalbumin and HFD induced mice model of obese asthma	Increase superoxide dismutase, glutathione reductase, and glutathione peroxidase activity	(Kleniewska and Pawliczak 2019)
Lipoic acid			
Probiotics			
Sakuranetin	Ovalbumin induced mice model of asthma	Inhibits the 8-iso-prostaglandin F2a in lung tissue	(Sakoda et al. 2016)
Astragalin	In vitro study on BEAS-2B	Inhibition of total ROS production and protein expression of PLCg1, PKCb2, NADPH oxidase subunits of p22^phox^, and p47^phox^	(Cho et al. 2014)
Morin	Ovalbumin induced mice model of asthma	Inhibition of total ROS production and phosphorylation of MAPK family protein (ERK, JNK, P38)	(Ma et al. 2016)
Resveratrol	Ovalbumin and HFD induced mice model of obesity-associated allergic pulmonary inflammation	Resveratrol reduced the p47phox and iNOS protein expression, ROS production, and elevated the SOD levels in lung tissues.	(André et al. 2016)

## Antioxidant scavenging system (Nrf2 pathway) in asthma

As discussed earlier in this manuscript, oxidative stress causes cellular dysfunction and abnormal release of toxic substances such as alcohols, aldehydes, peroxides, ketones, and cholesterol oxide (Finaud et al. 2006). Various factors are responsible for causing oxidative stress and inflammation in the lung such as ozone, diesel exhaust, and tobacco smoke. Oxidative stress is responsible for alteration in Th-1 and Th-2 that causes activation of NF-κB, which is a potential inducer for pro-inflammatory genes (Dozor 2010). The excess production of these ROS is controlled and co-ordinated by various endogenous antioxidant defense mechanisms which come into existence in the presence of exogenous irritants. Nrf2 is one such important transcription factor and a key regulator production of various cytoprotective proteins like antioxidants and xenobiotic detoxification enzymes to restore the balance between oxidant-antioxidant system (Sussan et al. 2015). Under normal conditions, Nrf2 is inactive in the cytoplasm bounds with its inhibitor Kelch-like ECH-associated protein (Keap1); however, in the presence of various environmental irritants, Nrf2 releases from the Keap1 and translocates to nucleus to induce the transcription of more than 650 proteins to activate different antioxidant pathways to attenuate the environmental irritant–mediated oxidative stress (Sussan et al. 2015).

**Fig. 7:**
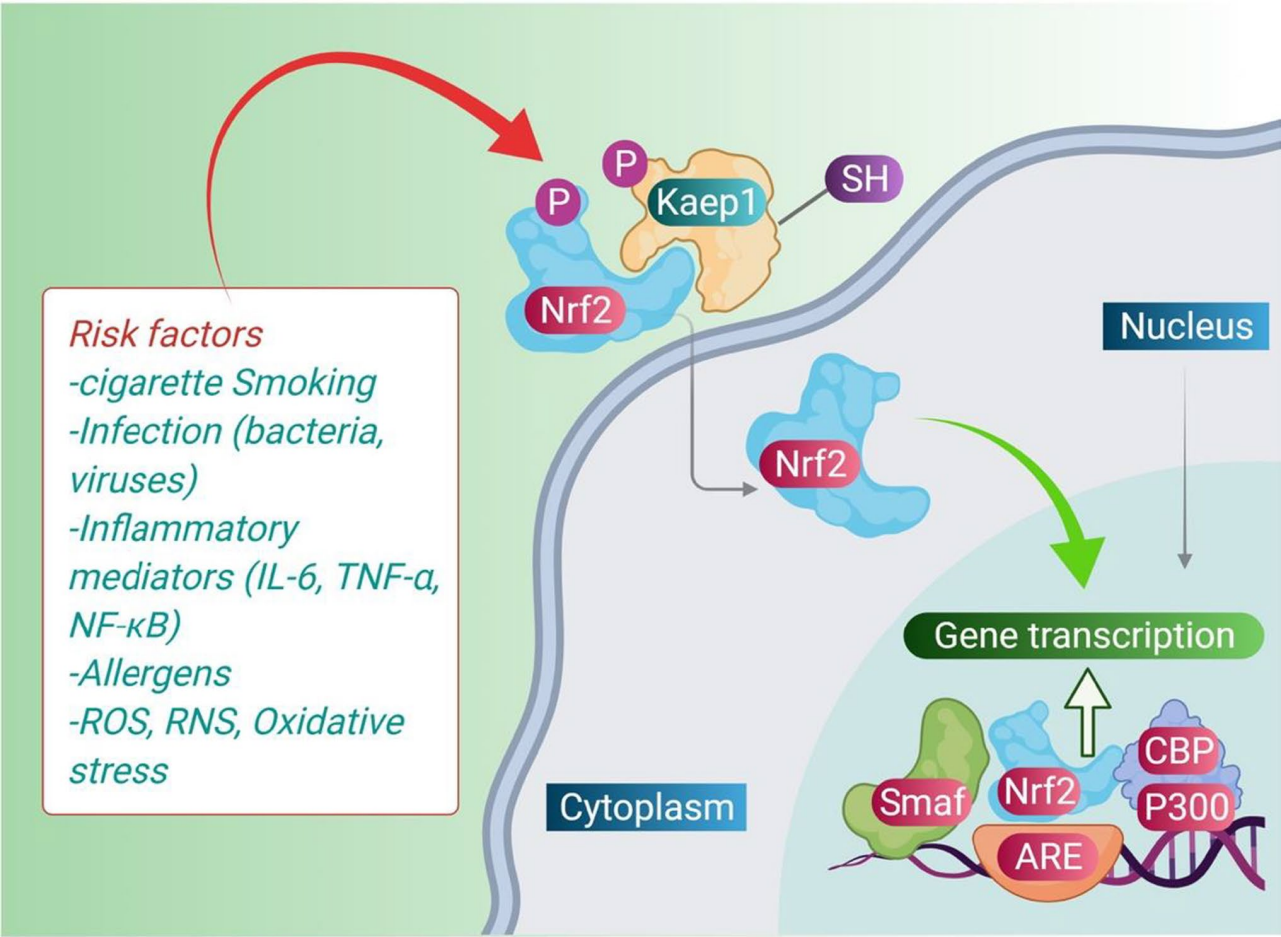
Mechanism of Nrf 2 in asthma. Nrf2, an antioxidant activator when combines with Kelch-like ECH-associated protein (Keap)1, undergoes phosphorylation. The phosphorylated Nrf2 translocates to the nucleus and combines with the ARE to induce transcription of the various antioxidative enzymes to restore the antioxidant system that was disrupted in the presence of various environmental irritants

Moreover, Nrf2 is also involved in various intracellular defense mechanisms to restore the airway epithelial barrier by repairing the disrupted epithelial junctions that occurred due to the exposure of the airways to various environmental triggers (Du et al. 2021). Aldehyde oxidase (AOX)1 acts through downregulation of Nrf2 pathways in the formation of the airway epithelial barrier. According to earlier data, therapeutics targeting the Nrf2/AOX1 pathway can reduce asthma by increasing airway epithelial barrier integrity (Mizumura et al. 2020). The detailed mechanism involving Nrf2 pathways in the pathogenesis of asthma is presented in Fig. 7.

Several antioxidants, those of which have shown positive impact against asthma and produced inhibitory effects against Nrf2 pathways, have been identified recently. In one of the studies, Wang et al. reported the effects of aloperine which suppresses allergic airway inflammation by altering the levels NF-κB, MAPK, and Nrf2/HO-1 pathways. Aloperine is reported to attenuate the NF-κB translocation factor and MAPK pathway in a murine asthma model and has activated Nrf2/HO-1 signaling pathway in asthmatic mice. It has also shown inhibitory effects on pro-inflammatory cytokines including IL-4, IL-5, IL-13, and IFN-γ, and IgE. The findings suggested that aloperine has potent anti-inflammatory and antioxidant effects that may be employed in the treatment of asthma (Wang et al. 2018). In another study, Mishra et al. have reported effects of vitamin E and curcumin in the activation of Nrf2 pathways in rat’s heart under altered thyroid states. Combination of vitamin E and curcumin regulated the levels of Kelch ECH associating protein (KEAP1) as well as Nrf2 and enhanced their antioxidant potential (Mishra et al. 2019).

Jung et al. have investigated the anti-inflammatory and antioxidant effects of the ethanolic extract of the medicinal herb, *Scrophularia koraiensis Nakai* (SKNEE) in BALB/c mice for the treatment of asthma. The study revealed that SKNEE attenuated the level of NF-κB and activated Nrf-2, HO-1 signaling pathways. The results indicated that SKNEE is a potential therapeutic agent for allergic airway inflammation (Jung et al. 2020). In another study, Chen et al. reported anti-asthmatic effects of one of the Chinese herbal plants *Schisandrin B* in a mouse model*.* The study exposed the antioxidant and anti-inflammatory effects of *Schisandrin B* in OVA-induced allergic asthma. *Schisandrin B* suppressed the level of NF-κB and activated the level of Nrf2 signaling pathways. Dworski et al. have reported anti-asthmatic effects of vitamin E which activated Nrf2 signaling pathways. The study revealed that activated levels of Nrf2 signaling pathways downregulated the levels of SOD-1 which showed higher antioxidant properties (Dworski et al. 2011). Other nutraceutical bioactive substances having antioxidant properties are mentioned in Table 2.

**Table 2:** List of important nutraceuticals and their mode of action at molecular and cellular level in asthma

Nutraceuticals	Mode of action	Reference
Phycocyanobilin	Inhibits NADPH oxidase complexes	(McCarty 2007)
Lipoic acid	Inhibits airway inflammation and hyperresponsiveness	(Cho et al. 2004)
Glycine	NF-κB and NLRP3 inhibition of inflammasome signaling pathway	(Fogarty et al. 2004)
Selenium	Ability to inhibit the path of free radicals and reduces the degree of inflammation	(Norton and Hoffmann 2012)
Zinc	Immunomodulator and oxidative stress severity control	(Rerksuppaphol and Rerksuppaphol 2016)
Mg	Ability to improve calcium influx to activate myosin light chain kinase (MLCK)	(Ohki et al. 1997)
Citrulline	L-citrulline can improve asthma control by increasing S-nitrosoglutathione (GSNO), the major source of NO bioactivité in the lung. It reduces NOS2 decouplement and reduces nitrosating stress asthma controller	(Holguin et al. 2019)
Folate	Suppresses allergic reactions and reduces allergy and asthma severity	(Blatter et al. 2013)
Biotin	Promotes human natural killer (NK) lymphocytes, for the generation of cytotoxic T lymphocytes (CTLs)	(Agrawal et al. 2016)
n-acetylcystine (NAC)	Supports H2S biosynthesis	(Lee et al. 2020)
Glycine	Dilates bronchioles	(Comhair et al. 2015)
Vitamin A	Regulation and production of pro-inflammatory cytokines such as TNF-α at cellular level and its control	(Bansal et al. 2014)
Vitamin C	Mitigates bronchoconstriction caused by exercise in asthma and stimulates the immune system	(Bansal et al. 2014; Harada et al. 2015)
Vitamin E	Inhibits airway eosinophilia and mucus cell hyperplasia AHR and inhibits iNOS, prostaglandin E2, pro-inflammatory cytokines, cyclo-oxygenase-2, and NF-κB expression	(Harada et al. 2015)
Omega-3-(*n*-3) fatty acids: α-linolenic acid (ALA), eicosapentaenoic acid (EPA), and docosahexaenoic acid (DHA).	Protective effects against exercise-induced bronchoconstriction, and an inhibited release of pro-inflammatory cytokines	(Hodge et al. 1996)
Omega-6-(*n*-6) fatty acids: linoleic acid (LA), γ-linolenic acid (GLA), and arachidonic acid (ARA)	Mechanism remains to be elucidated	(Hodge et al. 1996)
Glutathione	GSH balances Th1/Th2 responses, modifies the metabolism of nitric oxide, and impedes ROS	(Ferrini et al. 2013)
Superoxide dismutase (SOD)	SOD protects from harmful ROS and inflammation of the airways	(Kim et al. 2015)
Glutathione peroxidases	Glutathione peroxidases prevent inflammation and destruction of the airways	(Shaheen et al. 2015)
Apocynin	Inhibits NADPH oxidase in airway inflammation	(Kim et al. 2012)
Naringenin	Inhibits airway inflammation by downregulating gene expression of IL-6, IL-8, IL-1β, TNF-α	(Chin et al. 2020; Wadhwa et al. 2021)

*LCNs*, liquid crystalline nanoparticles; *BEAS-2B*, human bronchoepithelial cell line; *ROS*, reactive oxygen species; *NADPH*, Nicotinamide adenine dinucleotide phosphate; *Nox*, NAPDH oxidase; *Nqo1*, NADPH dehydrogenase quinone 1; *GCLC*, glutamate-cysteine ligase catalytic subunit; *NO*, nitric oxide; *iNOS*, inducible nitric oxide synthase; *EPO*, eosinophil peroxidase; *HFD*, high fat diet; *PLCg1*: phospholipase C gamma 1; *PKCb2*, protein kinase C beta 2; *MAPK*, mitogen activated protein kinase; *ERK*, extracellular regulated terminal kinase; *JNK*, c-Jun N-terminal kinase; *SOD*, superoxide dismutase

## Nutraceuticals under clinical trials for asthma treatment

Various nutraceutical-based clinical trials have proven their beneficial role in the management of asthma. A randomized controlled trail (RCT) with *n* = 80 subjects was conducted to evaluate if saffron supplementation could improve the clinical symptoms of asthma and reduced the severity in patients with mild/moderate allergic asthma. Among two groups (saffron and placebo), the subjects receiving two capsule of saffron (100 mg/day) for 8 weeks showed improvement in the frequency of clinical symptoms measured in terms of shortness of breath during the day and night time (frequency), use of standard asthma medicine (salbutamol spray), waking up at night due to asthma exacerbation, and limitation in physical activity (Zilaee et al. 2019). Similarly, another RCT investigated if *Nigella sativa* supplement reduced airway inflammation and improved the lung function in partly controlled asthma patients. *N. sativa* was administered in two groups as 1 g/day (*n* = 26 patients) and 2 g/day (*n* = 26 patients) for 3 months. The effects were then compared with placebo control (*n* = 24 patients). Interestingly, forced expiratory volume (FEV) 25–75% and FEV1 (% predicted) was remarkably increased in *N. sativa* 2 g/day group while peak expiratory flow variability was improved in both 1 and 2 g/day groups as compared to placebo. In addition, fractional exhaled nitric oxide and (FENO) and serum IgE were reduced, and interferon gamma was increased after 3 months in both 1 and 2g/day groups. Furthermore, asthma control test score was improved drastically at 6 and 12 weeks suggesting *N. sativa* supplementation may improve set lung function parameters and airway inflammation in partly controlled asthma (Salem et al. 2017).

Another RCT studied if lycopene-rich supplement modified non-eosinophilic airway inflammation in asthma. Asthmatic subjects (*n* = 32) were administered with a low antioxidant diet for 10 days before starting the randomized cross over trial (1 week for each of 3 treatments; placebo, tomato extract (45 mg lycopene/day) and tomato juice (45 mg lycopene/day) with 10 days washout period after each treatment. It was observed that low antioxidant diet was associated with worsening of asthma control score, reduction in %FEV (1) and %FVC. It also caused an increase in percent of sputum neutrophil. In contrast, both treatment groups (tomato juice and extract) decreased the influx of airway neutrophil. Furthermore, treatment with tomato extract also reduced sputum neutrophil elastase activity thus suggesting that dietary antioxidants such as lycopene may be beneficial in asthma management (Wood et al. 2008). Clinical trials have also found that dietary intake of the soy isoflavone genistein for 4 weeks is associated with reduced severity of asthma (Kalhan et al. 2008). Genistein inhibited eosinophil leukotriene (LTC)-4 synthesis from human peripheral blood eosinophil and inhibited phosphorylation of p38 mitogen–activated protein kinase (MAPK) and its downstream target MAPKAP-2, which in turn reduced the translocation of 5-lipoxygenase to the nuclear membrane. In patients with asthma, following 4 weeks of dietary soy isoflavone supplementation, ex vivo eosinophil LTC-4 synthesis decreased by 33% (*N* = 11, *P* = 0.02) and FENO decreased by 18% (*N* = 13, *P* = 0.03). This clinical trial highlights that dietary soy isoflavone supplementation is beneficial in the management of eosinophilic asthma (Kalhan et al. 2008). Ascorbic acid (an antioxidant significantly present in citrus fruits) is well-known for its beneficial activity against a range of lung diseases including asthma (Riccioni et al. 2007). A randomized controlled trial conducted in 80 asthmatics found that ascorbic acid supplementation at a dose of 1500 mg/day for 2 weeks was able to attenuate exercise-induced bronchoconstriction. This was evident by significant reduction in the maximum fall in post-exercise FEV1 and improvement in asthma symptom score by ascorbic acid compared to placebo. Post-exercise FENO, LTC4-E4 and 9alpha, and 11beta-prostaglandin F2 concentrations were significantly decreased on the ascorbic acid diet compared to the placebo and usual diet (Tecklenburg et al. 2007). Some of the aforementioned nutraceuticals are already available commercially while some are involved in ongoing clinical trials. These are exploring the antioxidant potential of promising nutraceuticals. Taken together, these nutraceuticals with “drug-like” potentials and less side effect than synthetic compounds need further validation for efficacy and safety before gaining its therapeutic value in clinical settings.

## Conclusion and future perspectives

It is clear from this extensive literature that oxidative stress and the imbalance between the oxidant and antioxidant systems play a major role in the initation and progression of the asthma disease. As the imbalance between the oxidant and antioxidant systems is not controlled by the endogenous antioxidant systems, it is therefore highly essential to identify the exogenous antioxidant which can restore the balance. Thus, identifying newer pharmacological agents that could restore the balance between the oxidant and antioxidant systems will become an alternate to the current therapy which are being used with limitations due to the poor response and adverse effects. Although various studies have reported on using the nutraceuticals as the alternative antioxidant therapy, which is quite promising in various in vitro and in vivo studies. Very few have been proven to be clinically effective in attenuating the disease due to poor bioavailability. Furthermore, extensive in-depth research is highly required to understand the new pathways in mediating the oxidative stress and at the same time identifying newer nutraceuticals with improved physiochemical properties which is essential in management of the asthma.

## Data Availability

No datasets were generated or analyzed during the current study.
